# (8-Hy­droxy-6-meth­oxy-1-oxo-1*H*-isochromen-3-yl)methyl formate: a supra­molecular framework

**DOI:** 10.1107/S2414314620013917

**Published:** 2020-10-23

**Authors:** Mustapha Tiouabi, Raphaël Tabacchi, Helen Stoeckli-Evans

**Affiliations:** a Institute of Chemistry, University of Neuchâtel, Av. de Bellevax 51, CH-2000 Neuchâtel, Switzerland; b Institute of Physics, University of Neuchâtel, rue Emile-Argand 11, CH-2000 Neuchâtel, Switzerland; Benemérita Universidad Autónoma de Puebla, México

**Keywords:** crystal structure, isocoumarin, cytogenin, hydrogen bonding, C—H⋯π inter­action, supra­molecular framework

## Abstract

In the title compound, C_12_H_10_O_6_, an intra­molecular O—H⋯O hydrogen bond forms an *S*(6) ring motif. The mol­ecule is essentially planar with an r.m.s. deviation of 0.051 Å for all non-H atoms. In the crystal mol­ecules are linked by C—H⋯O hydrogen bonds and a C—H⋯π inter­action, forming a supra­molecular framework.

## Abstract 2

In the crystal of the title compound, mol­ecules are linked by C—H⋯O hydrogen bonds and a C—H⋯π inter­action, forming a supra­molecular framework.

## Graphical abstract

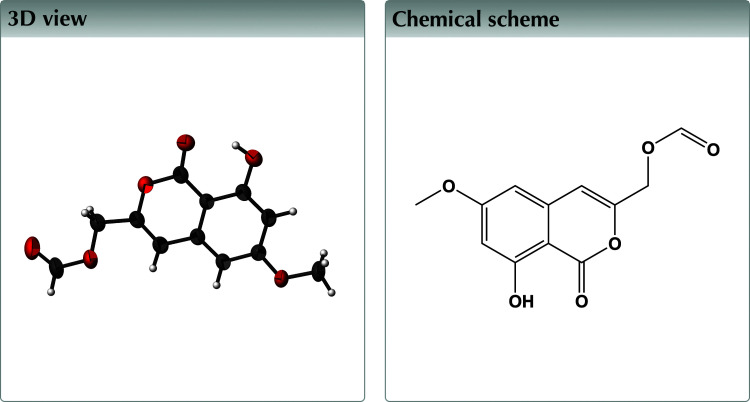

## Structure description

The title compound, **I**, is an inter­mediate in the synthesis of cytogenin, a naturally occurring isocoumarin that was first isolated from a cultured broth of *Streptoverticillium eurocidicum* (Kumagai *et al.*, 1990 ▸, 1995 ▸). It was shown by these authors to have both anti­biotic properties and anti­tumor activity. The first synthesis of cytogenin was reported in 2004 (Saeed, 2004 ▸). More recently, a new synthetic route to cytogenin and similar isocoumarins has been reported (Gadakh & Sudalai, 2014 ▸).

**Figure 1:**
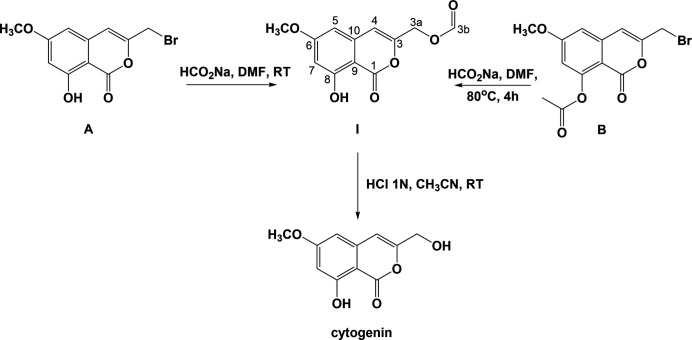
The reaction pathways for the synthesis of compound **I** and cytogenin (Tiouabi, 2005 ▸).

As shown in Fig. 1 ▸, compound **I** was prepared *via* two pathways (see *Synthesis and crystallization*). The details of the syntheses of the precursors **A** and **B** and cytogenin have been described elsewhere (Tiouabi, 2005 ▸).

**Figure 2:**
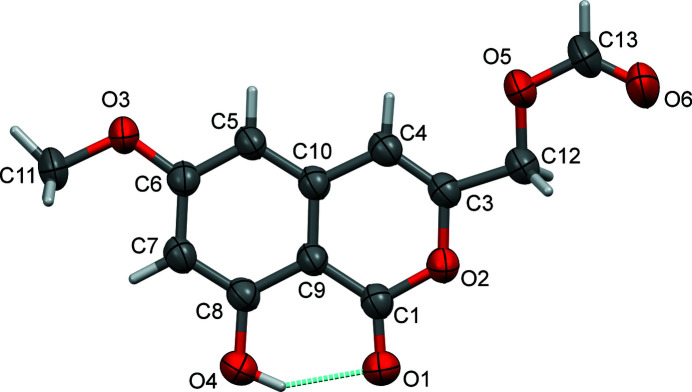
A view of the mol­ecular structure of compound **I**, with atom labelling. Displacement ellipsoids are drawn at the 50% probability level. The intra­molecular O—H⋯O hydrogen bond (see Table 1 ▸) is shown as a dashed line.

The mol­ecule of **I** (Fig. 2 ▸), is essentially planar with an r.m.s. deviation of 0.051 Å for all non-H atoms (O1–O6/C1/C3–C13); the maximum deviations from this mean plane are 0.080 (6) Å for atom C12 and −0.091 (8) Å for atom C13. There is an intra­molecular O—H⋯O hydrogen bond present, forming an *S*(6) ring motif (Fig. 2 ▸ and Table 1 ▸).

**Figure 3:**
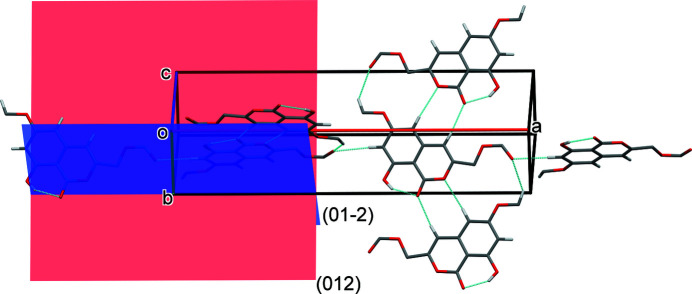
A partial view of the crystal packing of compound **I**, viewed normal to plane (011). Hydrogen bonds (see Table 1 ▸) are shown as dashed lines.

**Figure 4:**
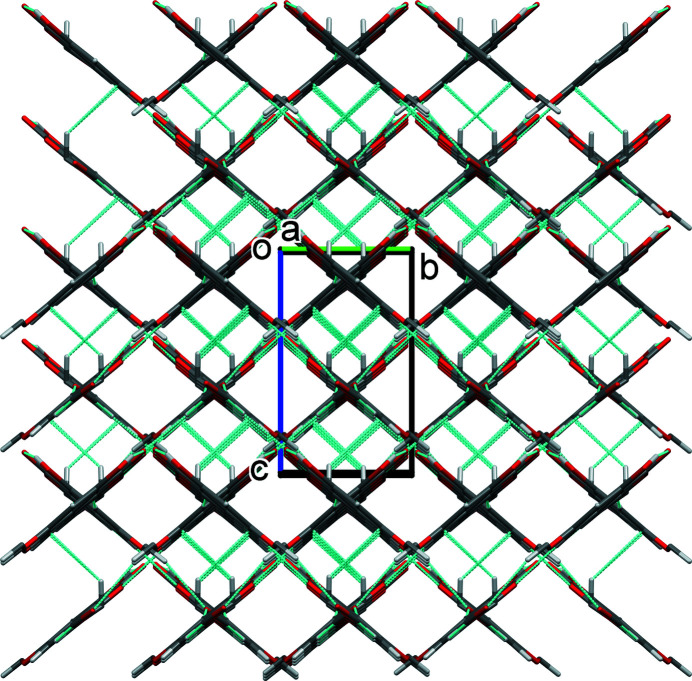
A view along the *a* axis of the crystal packing of compound **I**. Hydrogen bonds and C—H⋯π inter­actions (see Table 1 ▸) are shown as dashed lines.

In the crystal, mol­ecules are linked by a series of C—H⋯O hydrogen bonds (Table 1 ▸), forming inter­connected ribbons running normal to each other in planes (012) and (01 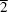 ): see Fig. 3 ▸. These inter­actions lead to the formation of a supra­molecular framework, which is reinforced by a C—H⋯π inter­action (Fig. 4 ▸ and Table 1 ▸).

## Synthesis and crystallization

The syntheses of the title compound, **I**, and cytogenin, are illustrated in Fig. 1 ▸. The syntheses of the precursors, 3-(bromo­meth­yl)-8-hy­droxy-6-meth­oxy-1*H*-isochromen-1-one (**A**), 3-(bromo­meth­yl)-6-meth­oxy-1-oxo-1*H*-isochromen-8-yl acetate (**B**), and cytogenin, are described in the PhD thesis of Tiouabi (2005 ▸), which can be downloaded from the website https://doc.rero.ch/record, a digital library where many theses of Swiss universities are deposited. The numbering scheme of **I** in Fig. 1 ▸ is with reference to the NMR spectra.

*Method 1*: The hy­droxy­bromo­isocoumarin **A** (0.14 g, 0.49 mmol) was dissolved with stirring in 5 ml of anhydrous DMF in a 50 ml flask equipped with a magnetic stirrer and under an atmosphere of argon. HCO_2_Na (0.167 g, 2.46 mmol) was added and the mixture was stirred overnight at room temperature. The evolution of the reaction was monitored by thin-layer chromatography, using di­methyl­chloride as eluent. On completion of the reaction, the mixture was diluted with ethyl acetate and then washed with an aqueous saturated solution of NaCl. The organic phase was dried using anhydrous Na_2_SO_4_, then filtered and concentrated using a rotary evaporator, yielding compound **I** in the form of a white solid (yield 0.118 g, 96%).

*Method 2*: The acet­oxy­bromo­isocoumarin **B** (34.2 mg, 0.104 mmol) was dissolved with stirring in 3 ml of anhydrous DMF in a 50 ml flask equipped with a magnetic stirrer and under an atmosphere of argon. HCO_2_Na (47 mg, 0.69 mmol) was added, the temperature was raised to 80°C and the mixture stirred for 4 h. The evolution of the reaction was monitored by thin-layer chromatography, using di­methyl­chloride as eluent. On completion of the reaction, the mixture was diluted with ethyl acetate and then washed with an aqueous saturated solution of NaCl. The organic phase was dried using anhydrous Na_2_SO_4_, then purified by column chromatography (silica, eluent CH_2_Cl_2_/hexane 10/1). Compound **I** was obtained in the form of a white solid (yield 18.7 mg, 72%).

*Analytical data for **I***: *R*_f_ (CH_2_Cl_2_, UV) 0.44. ^1^H NMR (400 MHz, CDCl_3_, 298 K): 3.90 (*s*, 3H, OCH_3_), 4.99 (*s*, 2H, CH2–3a), 6.42 (*d*, *J*_m_ = 2.3 Hz, 1H, ArH-7), 6.53 (*s*, 1H, H-4), 6.55 (*d*, *J*_m_ = 2.3 Hz, 1H, ArH-5), 8.17 (*s*, 1H, CHO-3 b), 11.0 (*s*, 1H, OH-8). ^13^C NMR (100 Hz, CDCl_3_, 298 K, HETCOR-SR/LR): 56.19 C(OCH_3_), 61.61 C(3a), 100.67 C(9), 101.80 C(5), 103.13 C(7), 107.82 C(4), 138.21 C(10), 150.27 C(3), 160.37 C(3 b), 164.23 C(1), 165.75 C(8), 167.35 C(6). HR–MS [ESI(+)]: ms 273.03634 [*M* + Na]^+^. IR (KBr disk, cm^−1^): 3129 *br*, 1728 *s*, 1690 *vs*, 1622 *m*, 1400 *vs*, 1164 *vs*, 1064 *w*.

Colourless block-like crystals of **I** were obtained by slow evaporation of a solution in chloro­form.

## Refinement

Crystal data, data collection and structure refinement details are summarized in Table 2 ▸. Intensity data were measured using a Stoe IPDS I, a one-circle diffractometer. The alert *diffrn_reflns_laue_measured_fraction_full value* (0.947) below minimum (0.95) is given. This involves 29 random reflections out of the expected 1034 for the IUCr cut-off limit of (sin θ)/λ = 0.6 Å^−1^; *viz*. 2.8%.

## Supplementary Material

Crystal structure: contains datablock(s) I, Global. DOI: 10.1107/S2414314620013917/bh4057sup1.cif

Structure factors: contains datablock(s) I. DOI: 10.1107/S2414314620013917/bh4057Isup2.hkl

Supporting information file. DOI: 10.1107/S2414314620013917/bh4057Isup3.cml

CCDC reference: 2039333

Additional supporting information:  crystallographic information; 3D view; checkCIF report

## Figures and Tables

**Table 1:** Hydrogen-bond geometry (Å, °) *Cg* is the centroid of the C5–C10 ring.

*D*—H⋯*A*	*D*—H	H⋯*A*	*D*⋯*A*	*D*—H⋯*A*
O4—H40⋯O1	0.84	1.88	2.616 (5)	146
C4—H4⋯O1^i^	0.95	2.38	3.326 (6)	173
C5—H5⋯O2^i^	0.95	2.59	3.541 (6)	176
C7—H7⋯O6^ii^	0.95	2.55	3.499 (5)	175
C11—H11*C*⋯O6^i^	0.98	2.57	3.388 (8)	141
C12—H12*B*⋯*Cg* ^iii^	0.99	2.88	3.788 (6)	153

Symmetry codes: (i) 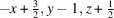 ; (ii) 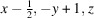 ; (iii) 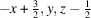 .

**Table 2:** Experimental details

Crystal data	
Chemical formula	C_12_H_10_O_6_
*M*_r_	250.20
Crystal system, space group	Orthorhombic, *P* *c* *a*2_1_
Temperature (K)	173
*a*, *b*, *c* (Å)	25.006 (2), 5.0337 (6), 8.5646 (6)
*V* (Å^3^)	1078.05 (17)
*Z*	4
Radiation type	Mo *K*α
μ (mm^−1^)	0.13
Crystal size (mm)	0.50 × 0.50 × 0.50

Data collection	
Diffractometer	STOE IPDS 1
Absorption correction	Multi-scan (*MULABS*; Spek, 2020 ▸)
*T*_min_, *T*_max_	0.763, 1.000
No. of measured, independent and observed [*I* > 2σ(*I*)] reflections	7630, 2012, 1249
*R*_int_	0.070
(sin θ/λ)_max_ (Å^−1^)	0.619

Refinement	
*R*[*F* ^2^ > 2σ(*F* ^2^)], *wR*(*F* ^2^), *S*	0.050, 0.135, 0.91
No. of reflections	2012
No. of parameters	166
No. of restraints	1
H-atom treatment	H-atom parameters constrained
Δρ_max_, Δρ_min_ (e Å^−3^)	0.32, −0.27

Computer programs: *EXPOSE*, *CELL* and *INTEGRATE* in *IPDS-I* (Stoe & Cie, 2004 ▸), *SHELXS97* (Sheldrick, 2008 ▸), *Mercury* (Macrae *et al.*, 2020 ▸), *SHELXL2018/3* (Sheldrick, 2015 ▸), *PLATON* (Spek, 2020 ▸) and *publCIF* (Westrip, 2010 ▸).

## full crystallographic data

### Crystal data

**Table:**

C_12_H_10_O_6_	*D*_x_ = 1.542 Mg m^−^^3^
*M**_r_* = 250.20	Mo *K*α radiation, λ = 0.71073 Å
Orthorhombic, *P**c**a*2_1_	Cell parameters from 3909 reflections
*a* = 25.006 (2) Å	θ = 2.2–25.8°
*b* = 5.0337 (6) Å	µ = 0.13 mm^−^^1^
*c* = 8.5646 (6) Å	*T* = 173 K
*V* = 1078.05 (17) Å^3^	Block, colorless
*Z* = 4	0.50 × 0.50 × 0.50 mm
*F*(000) = 520	

### Data collection

**Table:**

STOE IPDS 1 diffractometer	2012 independent reflections
Radiation source: fine-focus sealed tube	1249 reflections with *I* > 2σ(*I*)
Plane graphite monochromator	*R*_int_ = 0.070
φ rotation scans	θ_max_ = 26.1°, θ_min_ = 2.9°
Absorption correction: multi-scan (MULABS; Spek, 2020)	*h* = −30→30
*T*_min_ = 0.763, *T*_max_ = 1.000	*k* = −6→6
7630 measured reflections	*l* = −9→9

### Refinement

**Table:**

Refinement on *F*^2^	Secondary atom site location: difference Fourier map
Least-squares matrix: full	Hydrogen site location: inferred from neighbouring sites
*R*[*F*^2^ > 2σ(*F*^2^)] = 0.050	H-atom parameters constrained
*wR*(*F*^2^) = 0.135	*w* = 1/[σ^2^(*F*_o_^2^) + (0.0852*P*)^2^] where *P* = (*F*_o_^2^ + 2*F*_c_^2^)/3
*S* = 0.91	(Δ/σ)_max_ < 0.001
2012 reflections	Δρ_max_ = 0.32 e Å^−^^3^
166 parameters	Δρ_min_ = −0.27 e Å^−^^3^
1 restraint	Extinction correction: (SHELXL-2018/3; Sheldrick, 2015), Fc^*^=kFc[1+0.001xFc^2^λ^3^/sin(2θ)]^-1/4^
Primary atom site location: structure-invariant direct methods	Extinction coefficient: 0.061 (11)

### Special details

**Table:**

Geometry. All esds (except the esd in the dihedral angle between two l.s. planes) are estimated using the full covariance matrix. The cell esds are taken into account individually in the estimation of esds in distances, angles and torsion angles; correlations between esds in cell parameters are only used when they are defined by crystal symmetry. An approximate (isotropic) treatment of cell esds is used for estimating esds involving l.s. planes.
Refinement. Flack x = 0.223 (999) by classical fit to all intensities 1.664 (999) from 481 selected quotients (Parsons' method) ** Absolute structure cannot be determined reliably ** The hydroxyl H atom and the C-bound H atoms were included in calculated positions and treated as riding on their parent O or C atoms.

### Fractional atomic coordinates and isotropic or equivalent isotropic displacement parameters (Å^2^)

**Table:**

	*x*	*y*	*z*	*U*_iso_*/*U*_eq_	
O1	0.68528 (13)	0.9547 (7)	−0.0918 (5)	0.0500 (9)	
O2	0.75876 (11)	0.7819 (6)	0.0089 (4)	0.0461 (9)	
O3	0.58898 (11)	0.0468 (7)	0.3437 (5)	0.0511 (10)	
O4	0.59095 (13)	0.7730 (7)	−0.0142 (5)	0.0531 (10)	
H40	0.613134	0.862673	−0.065697	0.080*	
O5	0.86682 (12)	0.4224 (8)	0.1774 (5)	0.0657 (12)	
O6	0.94909 (14)	0.5664 (9)	0.1136 (6)	0.0732 (13)	
C1	0.70366 (17)	0.7848 (9)	−0.0037 (6)	0.0433 (11)	
C3	0.78373 (18)	0.5948 (9)	0.1006 (7)	0.0428 (12)	
C4	0.75774 (17)	0.4136 (9)	0.1838 (6)	0.0443 (12)	
H4	0.776871	0.288244	0.245052	0.053*	
C5	0.67032 (17)	0.2286 (9)	0.2657 (6)	0.0438 (11)	
H5	0.687823	0.104223	0.331620	0.053*	
C6	0.61467 (17)	0.2302 (9)	0.2549 (7)	0.0434 (12)	
C7	0.58804 (17)	0.4147 (10)	0.1602 (7)	0.0455 (12)	
H7	0.550100	0.414965	0.154550	0.055*	
C8	0.61731 (18)	0.5950 (9)	0.0759 (6)	0.0437 (13)	
C9	0.67425 (17)	0.5970 (9)	0.0848 (7)	0.0399 (11)	
C10	0.69987 (16)	0.4093 (9)	0.1799 (7)	0.0411 (11)	
C11	0.53177 (17)	0.0277 (11)	0.3310 (8)	0.0614 (15)	
H11C	0.518847	−0.116607	0.397911	0.092*	
H11B	0.521957	−0.008880	0.222346	0.092*	
H11A	0.515514	0.195648	0.364158	0.092*	
C12	0.84306 (18)	0.6344 (10)	0.0911 (7)	0.0493 (13)	
H12B	0.855082	0.629778	−0.019002	0.059*	
H12A	0.853189	0.807981	0.136752	0.059*	
C13	0.92037 (19)	0.4136 (14)	0.1770 (10)	0.0726 (19)	
H13	0.936777	0.272176	0.232693	0.087*	

### Atomic displacement parameters (Å^2^)

**Table:**

	*U*^11^	*U*^22^	*U*^33^	*U*^12^	*U*^13^	*U*^23^
O1	0.0486 (18)	0.0487 (18)	0.053 (2)	0.0019 (15)	−0.0012 (16)	0.0089 (19)
O2	0.0381 (16)	0.0448 (17)	0.055 (2)	−0.0039 (13)	−0.0010 (15)	0.0043 (17)
O3	0.0330 (15)	0.0564 (19)	0.064 (3)	−0.0051 (14)	0.0021 (16)	0.0138 (19)
O4	0.0425 (17)	0.055 (2)	0.062 (3)	0.0046 (16)	−0.0054 (17)	0.0109 (19)
O5	0.0318 (16)	0.076 (2)	0.089 (3)	−0.0021 (17)	−0.002 (2)	0.026 (2)
O6	0.040 (2)	0.099 (3)	0.080 (3)	−0.012 (2)	0.0013 (19)	0.016 (3)
C1	0.036 (2)	0.046 (3)	0.048 (3)	0.0014 (19)	0.001 (2)	−0.001 (2)
C3	0.035 (2)	0.045 (2)	0.048 (3)	0.003 (2)	−0.002 (2)	−0.002 (2)
C4	0.038 (2)	0.044 (2)	0.051 (3)	0.0004 (19)	−0.004 (2)	−0.002 (2)
C5	0.034 (2)	0.044 (2)	0.053 (3)	−0.001 (2)	−0.004 (2)	0.002 (2)
C6	0.038 (2)	0.039 (2)	0.053 (3)	−0.0005 (19)	0.002 (2)	0.001 (2)
C7	0.035 (2)	0.048 (2)	0.053 (4)	−0.003 (2)	−0.003 (2)	−0.002 (2)
C8	0.036 (2)	0.044 (3)	0.052 (4)	0.003 (2)	−0.002 (2)	0.001 (2)
C9	0.033 (2)	0.038 (2)	0.049 (3)	0.0002 (19)	−0.001 (2)	0.001 (2)
C10	0.035 (2)	0.041 (2)	0.048 (3)	0.0008 (18)	0.000 (2)	−0.001 (2)
C11	0.031 (2)	0.073 (3)	0.080 (4)	−0.011 (2)	−0.001 (3)	0.013 (3)
C12	0.036 (2)	0.055 (3)	0.057 (4)	0.000 (2)	−0.002 (2)	0.005 (3)
C13	0.031 (3)	0.088 (4)	0.098 (6)	0.000 (3)	−0.003 (3)	0.021 (4)

### Geometric parameters (Å, º)

**Table:**

O1—C1	1.230 (6)	C5—C10	1.383 (7)
O2—C3	1.376 (6)	C5—C6	1.395 (6)
O2—C1	1.382 (5)	C5—H5	0.9500
O3—C6	1.358 (6)	C6—C7	1.401 (7)
O3—C11	1.438 (5)	C7—C8	1.371 (7)
O4—C8	1.354 (6)	C7—H7	0.9500
O4—H40	0.8400	C8—C9	1.426 (6)
O5—C13	1.340 (6)	C9—C10	1.402 (7)
O5—C12	1.428 (6)	C11—H11C	0.9800
O6—C13	1.184 (7)	C11—H11B	0.9800
C1—C9	1.417 (7)	C11—H11A	0.9800
C3—C4	1.327 (7)	C12—H12B	0.9900
C3—C12	1.499 (6)	C12—H12A	0.9900
C4—C10	1.447 (6)	C13—H13	0.9500
C4—H4	0.9500		
			
C3—O2—C1	120.3 (4)	O4—C8—C9	120.8 (4)
C6—O3—C11	118.3 (4)	C7—C8—C9	120.6 (4)
C8—O4—H40	109.5	C10—C9—C1	121.5 (4)
C13—O5—C12	116.1 (4)	C10—C9—C8	118.8 (4)
O1—C1—O2	115.3 (4)	C1—C9—C8	119.6 (4)
O1—C1—C9	126.7 (4)	C5—C10—C9	120.5 (4)
O2—C1—C9	117.9 (4)	C5—C10—C4	122.1 (4)
C4—C3—O2	123.7 (4)	C9—C10—C4	117.4 (4)
C4—C3—C12	127.2 (5)	O3—C11—H11C	109.5
O2—C3—C12	109.1 (4)	O3—C11—H11B	109.5
C3—C4—C10	119.2 (5)	H11C—C11—H11B	109.5
C3—C4—H4	120.4	O3—C11—H11A	109.5
C10—C4—H4	120.4	H11C—C11—H11A	109.5
C10—C5—C6	119.6 (5)	H11B—C11—H11A	109.5
C10—C5—H5	120.2	O5—C12—C3	106.5 (4)
C6—C5—H5	120.2	O5—C12—H12B	110.4
O3—C6—C5	115.5 (4)	C3—C12—H12B	110.4
O3—C6—C7	123.4 (4)	O5—C12—H12A	110.4
C5—C6—C7	121.1 (4)	C3—C12—H12A	110.4
C8—C7—C6	119.3 (4)	H12B—C12—H12A	108.6
C8—C7—H7	120.3	O6—C13—O5	125.8 (6)
C6—C7—H7	120.3	O6—C13—H13	117.1
O4—C8—C7	118.6 (4)	O5—C13—H13	117.1
			
C3—O2—C1—O1	−178.0 (4)	O2—C1—C9—C8	179.5 (4)
C3—O2—C1—C9	2.3 (6)	O4—C8—C9—C10	179.9 (5)
C1—O2—C3—C4	−1.6 (7)	C7—C8—C9—C10	0.8 (8)
C1—O2—C3—C12	179.1 (4)	O4—C8—C9—C1	−1.0 (8)
O2—C3—C4—C10	−0.2 (8)	C7—C8—C9—C1	179.9 (5)
C12—C3—C4—C10	179.0 (5)	C6—C5—C10—C9	1.3 (8)
C11—O3—C6—C5	−176.5 (5)	C6—C5—C10—C4	−178.7 (5)
C11—O3—C6—C7	4.8 (8)	C1—C9—C10—C5	179.7 (5)
C10—C5—C6—O3	−179.7 (5)	C8—C9—C10—C5	−1.2 (8)
C10—C5—C6—C7	−1.0 (8)	C1—C9—C10—C4	−0.3 (8)
O3—C6—C7—C8	179.2 (5)	C8—C9—C10—C4	178.8 (5)
C5—C6—C7—C8	0.6 (8)	C3—C4—C10—C5	−178.9 (5)
C6—C7—C8—O4	−179.6 (5)	C3—C4—C10—C9	1.1 (8)
C6—C7—C8—C9	−0.5 (8)	C13—O5—C12—C3	176.8 (5)
O1—C1—C9—C10	179.0 (5)	C4—C3—C12—O5	5.5 (8)
O2—C1—C9—C10	−1.4 (7)	O2—C3—C12—O5	−175.2 (4)
O1—C1—C9—C8	−0.1 (8)	C12—O5—C13—O6	0.9 (12)

### Hydrogen-bond geometry (Å, º)

*Cg* is the centroid of the C5–C10 ring.

**Table:**

*D*—H···*A*	*D*—H	H···*A*	*D*···*A*	*D*—H···*A*
O4—H40···O1	0.84	1.88	2.616 (5)	146
C4—H4···O1^i^	0.95	2.38	3.326 (6)	173
C5—H5···O2^i^	0.95	2.59	3.541 (6)	176
C7—H7···O6^ii^	0.95	2.55	3.499 (5)	175
C11—H11*C*···O6^i^	0.98	2.57	3.388 (8)	141
C12—H12*B*···*Cg*^iii^	0.99	2.88	3.788 (6)	153

Symmetry codes: (i) −*x*+3/2, *y*−1, *z*+1/2; (ii) *x*−1/2, −*y*+1, *z*; (iii) −*x*+3/2, *y*, *z*−1/2.
